# Reliability, failure probability, and strength of resin-based materials for CAD/CAM restorations

**DOI:** 10.1590/1678-775720150561

**Published:** 2016

**Authors:** Kiatlin Lim, Adrian U-Jin Yap, Shruti Vidhawan Agarwalla, Keson Beng-Choon Tan, Vinicius Rosa

**Affiliations:** 1 Faculty of Dentistry, National University of Singapore, Singapore; 2 JurongHealth Services, Department of Dentistry, Singapore

**Keywords:** Tensile strength, Reliability and validity, Composite resins, Dental restoration failure, Dental porcelain

## Abstract

**Objective:**

This study investigated the Weibull parameters and 5% fracture probability of direct, indirect composites, and CAD/CAM composites.

**Material and Methods:**

Discshaped (12 mm diameter x 1 mm thick) specimens were prepared for a direct composite [Z100 (ZO), 3M-ESPE], an indirect laboratory composite [Ceramage (CM), Shofu], and two CAD/CAM composites [Lava Ultimate (LU), 3M ESPE; Vita Enamic (VE), Vita Zahnfabrik] restorations (n=30 for each group). The specimens were polished, stored in distilled water for 24 hours at 37°C. Weibull parameters (m= modulus of Weibull, σ0= characteristic strength) and flexural strength for 5% fracture probability (σ5%) were determined using a piston-on-three-balls device at 1 MPa/s in distilled water. Statistical analysis for biaxial flexural strength analysis were performed either by both one-way ANOVA and Tukey's *post hoc* (α=0.05) or by Pearson's correlation test.

**Results:**

Ranking of m was: VE (19.5), LU (14.5), CM (11.7), and ZO (9.6). Ranking of σ0 (MPa) was: LU (218.1), ZO (210.4), CM (209.0), and VE (126.5). σ5% (MPa) was 177.9 for LU, 163.2 for CM, 154.7 for Z0, and 108.7 for VE. There was no significant difference in the m for ZO, CM, and LU. VE presented the highest m value and significantly higher than ZO. For σ0 and σ5%, ZO, CM, and LU were similar but higher than VE.

**Conclusion:**

The strength characteristics of CAD/ CAM composites vary according to their composition and microstructure. VE presented the lowest strength and highest Weibull modulus among the materials.

## INTRODUCTION

Resin composites are widely used as direct posterior restorative materials due to good aesthetic, biological and physico-mechanical properties^16,22,24^. Resin composites used for direct restorations can present high elastic modulus and fracture strength. In addition, these materials can have a subcritical crack growth susceptibility coefficient as high as 40, resulting in high resistance to slow crack growth^15,16,24^. However, they have some inherent drawbacks such as polymerisation shrinkage, shrinkage stress, and limited depth of cure. Despite the advances in material technology, bulk fracture persists as one of the major causes of direct composite restoration failure in clinical trials^12,24^.

To overcome some of these limitations, indirect resin composites were introduced^14^. The polymerization protocols of these materials are optimized and often include the use of powerful lights for an extended period of time to achieve high degrees of conversion^4,14,21^. Furthermore, some can be heated to temperatures above the composite's glass transition temperature to allow an increase in polymer chain mobility, leading to additional cross-linking and stress relief^4,14,25^. Nonetheless, fabrication of indirect composites involves additional laboratory procedures that potentially increase the time required to deliver the restoration to the patient and operational cost. These disadvantages are reduced with CAD/CAM workflows and the introduction of CAD/CAM composite blocks^13^. CAD/ CAM fabrication of dental restorations simplifies handling and is less labour intensive, increasing efficiency at a reduced cost^5,13^.

Several resin-based CAD/CAM composites blocks are available with different compositions and microstructure [(e.g. polymer-infiltrated ceramic-network (Enamic) and nanoparticle and nanocluster-filled resin (Lava Ultimate Restorative)]. Although these two materials exhibit smoother milled margins compared to ceramic-based blocks^3^, they present lower flexural modulus, fracture toughness, and flexural strength than glass-ceramic and ceramic materials^3,11,20^.

The measure of structural performance for brittle dental materials is often estimated by their fracture strength values. However, structural performance cannot be directly predicted by strength data, since the latter is a contingent and not an inherent material property^17^. Weibull statistical analysis is largely applied for describing the scatter in fracture strength measurements of brittle materials. It is related to fracture probability and can describe the reliability of materials (e.g. stress required to fracture a given percentage of specimens). In addition, it can be scaled-up considering different specimen size (bulk volume or surface area under stress)^8,17^. The two-parameter Weibull distribution is based on two distinct parameters: Weibull modulus and characteristic strength. The Weibull modulus (m) is a dimensionless material-specific parameter that describes the variation in the strength or asymmetric strength distribution as a result of flaws within the microstructure^17^. Thus, low Weibull modulus values indicate higher scattering for fracture strength data and hence lower reliability. Since it is inversely related to the standard deviation in a normal distribution, high Weibull modulus relates to higher reliability of materials. A higher Weibull modulus equates to a more homogeneous flaw distribution throughout the entire volume, which results in higher structural reliability and lower failure probability^8,16,17,19^.

The objective of this study was to evaluate the biaxial flexural strength and Weibull parameters of CAD/CAM resin-based composites. The hypothesis to be tested is that CAD/CAM materials present higher strength features as compared with direct and indirect composite materials.

## MATERIAL AND METHODS

**Figure 1:**
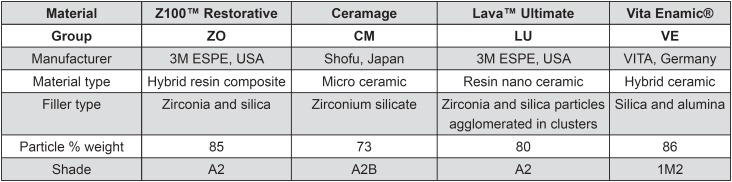
Characteristics of direct (Z0) and indirect (CM, LU and VE) resin composites according to the manufacturers

The materials studied are listed in Figure 1.

Disk shaped specimens (12 mm diam x 1 mm thick) were prepared as follows:

ZO and CM: specimens were prepared by condensing ZO and CM composites into a cylindrical metal mould. A glass slide was used to extrude excess material. ZO specimens were polymerised using a LED light unit (Monitex BlueLEX™ GT-1200, New Taipei City, Taiwan) at 600 mW/cm^2^ for 40 seconds *per* quadrant. For CM, the uncured discs were placed in a laboratory curing unit (Solidilite V, Shofu, Kyoto, Kyoto Prefecture, Japan) containing four halogen light bulbs of 150 W each and cured for 5 minutes.

LU and VE: a digital impression (CEREC Omnicam, Sirona, Charlotte, NC, USA) was obtained from a metal cylinder (12 mm in diameter x 14 mm in height) and used as a template to mill the blocks (CEREC inLab MC XL milling unit, Sirona, NC, USA). The cylindrical blocks were sliced into 1.1 mm thick discs (IsoMet 1000 Precision Cutter, Buehler, Bergneustadt, Cologne, Germany).

All the specimens were polished down (Buehler Phoenix Beta Grinder Polisher, Bergneustadt, Cologne, Germany) using a series of sand paper disc of decreasing grits (MicroCut discs, Germany: P1200, P2500 and P4000, 100 rpm / 30 seconds per grit paper) to 1 mm. The final dimensions of specimens were measured at four points diagonally using a digital calliper to ensure a final thickness of 1 mm and 12 mm diameter. Specimens were subsequently stored for 24 hours in distilled water at 37°C prior to testing.

The biaxial flexural strength of 30 specimens of each material was tested using a piston-on-three-balls device according to Ornaghi, et al.^17^ (2012) at a loading rate of 1 MPa/s. The biaxial flexural strength (σ_BI_) was calculated according to Equations 1 to 3, where P is the fracture load (in N); b is the thickness of the specimen at fracture origin (in mm); r1 is the radius of the support ball circle (5 mm); r2 is the radius of loaded area (0.6 mm); and r3 is the radius of the specimen (in mm). Poisson's ratio (u) was 0.3 for ZO and CM, 0.43 for LU and 0.23 for VE^6,16^.

Equation 1
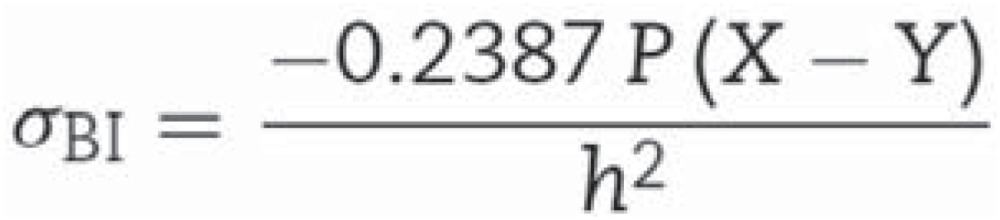


Equation 2
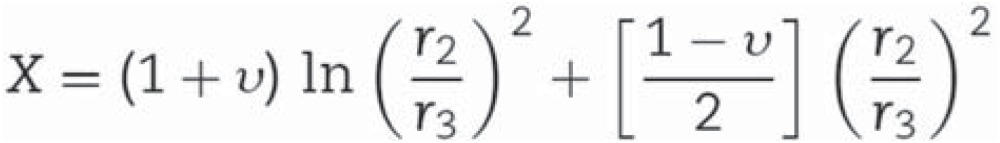


Equation 3
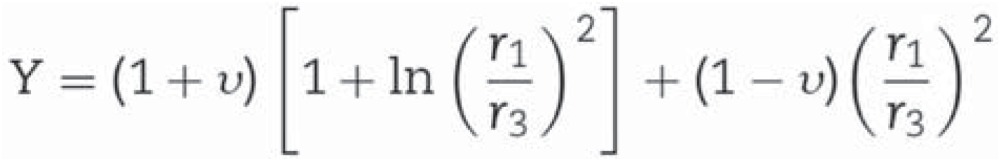


Fracture strength was fitted to the two-parameter Weibull distribution, which is comprised of Weibull modulus (m) and characteristic strength (σ_0_) obtained according to Equation 4

Equation 4
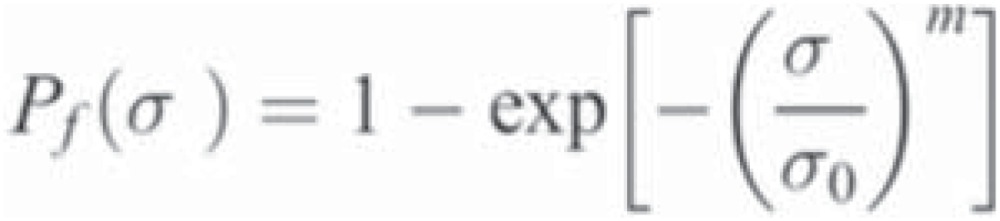


where Pf is the fracture probability. The parameter σ_0_ corresponds to the strength at the failure probability of 63.2%. Based on Equation 4, the parameter σ_5%_ was also determined, which corresponds to the strength at the more clinically relevant failure probability of 5%. The Weibull parameters were calculated based on the maximum likelihood method, according to the ASTM C 1239^2^ and plotted in addition to their 95% confidence intervals. Fractured surfaces of randomly selected specimens were examined using optical and scanning electron microscopy (Stereoscan 400, Wetzlar, Giessen, Germany).

Biaxial flexural strength and number of fragments were analyzed by one-way ANOVA and multiple comparisons were performed using Tukey's *post hoc* test at a pre-set significance level of 5%. Correlation between flexural strength and number of fragments were calculated using Pearson's correlation test.

## RESULTS

**Figure 2:**
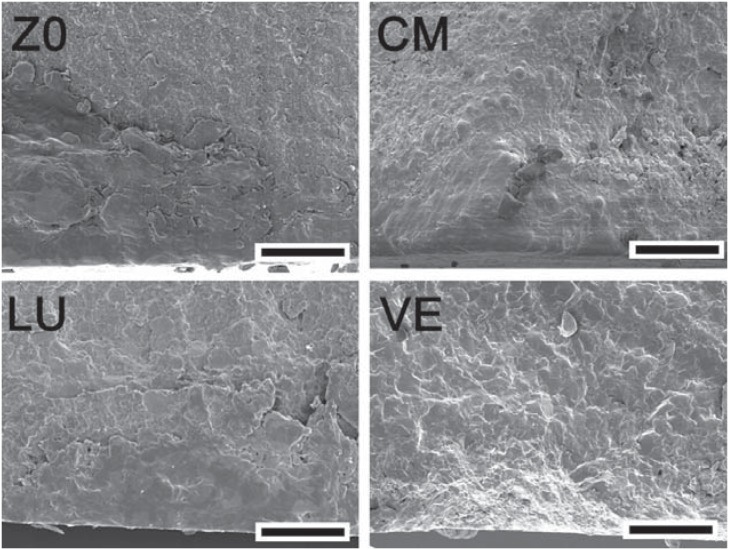
Fracture surfaces of the materials tested (scale bar= 500 μm)

**Figure 3:**
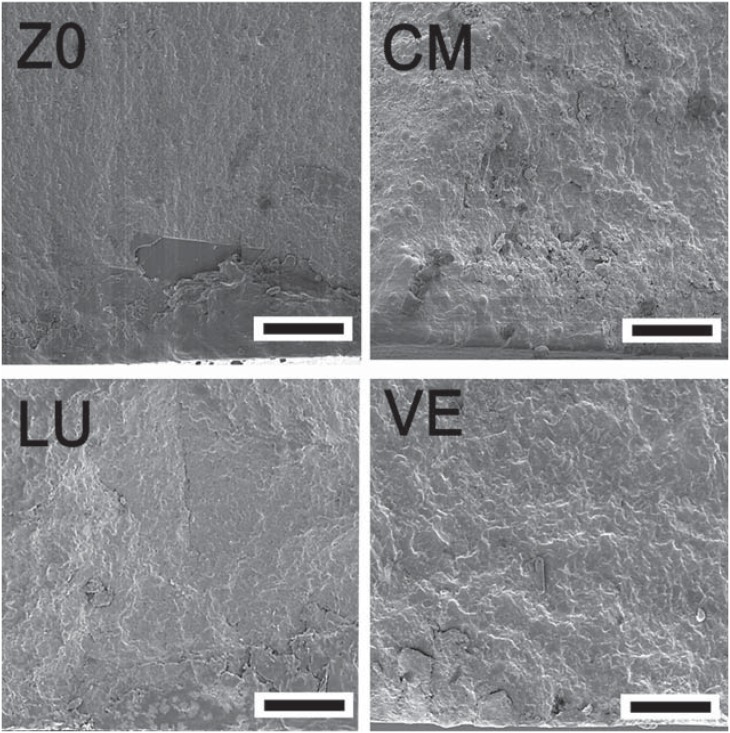
Fracture surfaces of the materials tested (scale bar= 25 μm)

The s_BI_ obtained for ZO, CM, and LU were similar, but significantly higher than VE (Table 1). There was a positive correlation between s_BI_ and the number of fragments. For ZO and CM, 40 and 43% of the specimens fractured into four pieces and for LU and VE, 57 and 83% of the discs produced three and two fragments, respectively. Figure 2 and 3 show the fracture surface of selected specimens.

**Table 1:** Mean biaxial flexural strength, MPa (SD), mean number of fractured fragments (SD) and Pearson correlation coefficient. Groups with similar superscript letter are not statistically different (p<0.05)

Group	sBI(MPa)	Number of fragments	Pearson correlation coefficient (r)
ZO	199±26^a^	4.4±0.9^a^	0.65
CM	200±21^a^	4.2±1.0^a^	0.494
LU	210±18^a^	3.3±0.5^b^	0.609
VE	129±22^b^	2.2 ±0.4^c^	0.761

**Figure 4:**
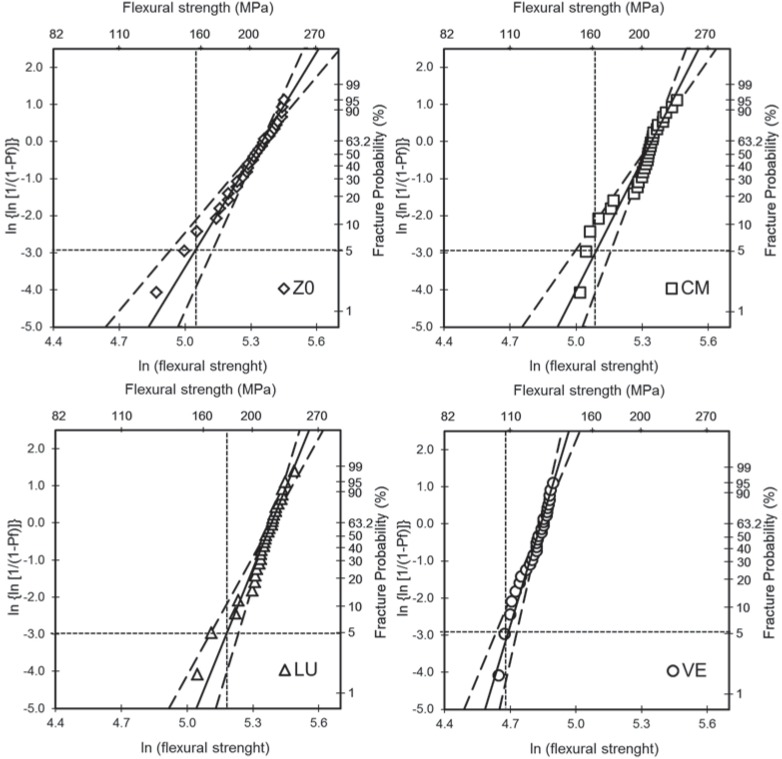
Weibull plot of the materials tested. Dashed lines indicate the 95% confidence interval of the Weibull modulus. Dotted line indicate flexural strength at 5% fracture probability (σ5%)

Table 2 shows the Weibull parameters and the respective 95% confidence intervals. The Weibull plots are shown in Figure 4. There was no significant difference in the m for ZO, CM, and LU as the confidence intervals overlap. VE presented the highest m value and significantly higher than ZO. For σ_0_ and σ_5%_, ZO, CM, and LU were similar, but higher than VE.

**Table 2:** Weibull parameters (Weibull modulus, m; characteristic strength, σ_0_), flexural strength at 5% failure probability (σ5%), and their respective 95% confidence intervals in brackets. Groups with similar superscript letter are not statistically different (p<0.05).

Group	m	σ(MPa)	σ5% (MPa)
Z0	9.6 (7.0 – 13.0)^b^	210.4 (201.4 – 219.6)^a^	154.7 (137.8– 167.7)
CM	11.7 (8.5 – 15.8)^b^	209.0 (201.5 – 216.5)^a^	163.2 (147.4 – 173.4)
LU	14.5 (10.6 – 19.5)^b^	218.1 (211.9 – 224.2)^a^	177.9 (165.0 – 187.4)
VE	19.5 (14.1 – 26.4)^a^	126.5 (123.8 – 129.2)^b^	108.7 (102.6 – 113.1)

There was an average positive correlation between σ_BI_ and the number of fragments for all the materials tested (Table 1).

## DISCUSSION

The hypothesis was rejected, since LU presented similar strength characteristics as CE and ZO. Table 1 shows that the biaxial strength of VE was significantly lower than the other materials tested. Similar trends have been previously reported for flexural strength of VE^3,10^. VE is composed of a porous feldspathic ceramic matrix, infiltrated with a urethane dimethacrylate and triethylene glycol dimethacrylate copolymer. This combination increases the ability to endure mechanical loading by experiencing more elastic deformation before failure^3^. This can possibly be the reason why VE fractured in fewer fragments as compared with the other materials tested (Table 1).

Brittle materials, such as ceramics and composites, are very sensitive to the presence of flaws^15,19^. These flaws act as stress concentrators and are often characterized as scratches, non-uniform matrix and filler interface, foreign bodies, pores, and other innate defects. In direct restorative materials, they can be introduced during the manufacturing process, clinical handling, fabrication or finishing of restorations^16^. The fractographic analysis showed that, typically, the fractures were originated from surface flaws of semi-elliptical shape (Figure 2 and 3). Similar critical defects have been previously observed on fracture surfaces porcelains and resin composites^18,19^. Interestingly, the Weibull modulus (m) for LU was slightly higher than that obtained for ZO and CM. As the procedures involved to fabricate LU specimens were automated, it may be suggested that the handling of these materials had a minor effect in adding defects to the pre-existing flaw population.

The characteristic strength (σ_0_) corresponds to the stress level for a 63.2% probability of failure. This location parameter is dependent on the stress configuration and test specimen size. The σ_0_ obtained for Z0, CM, and LU were similar (Table 2) and at least 65% higher than the one obtained for VE. Nonetheless, it presented the highest m, resulting in a material with the utmost reliability and predictability for failure among the materials tested. The existence of the polymer phase within the ceramic framework spread the plasticity upon loading, hence increasing the crack resistance with crack extension. This could give a higher observable Weibull modulus, rather than toughening itself, resulting in a material with high m and moderate σ_0_^7,9,11^.

At a more clinically relevant failure rate (σ_5%_), LU presented the highest value, followed by CM and ZO respectively (Table 2). The higher strength for LU may be related to its microstructure, which is composed of 0.6-1.0 μm aggregated zirconia/ silica clusters (consisting of 20 nm silica and 4-11 nm zirconia particles); non-agglomerated/non-aggregated 20 nm silica nanoparticles; and non-agglomerated/non-aggregated 4 to 11 nm zirconia nanoparticles^1^. The wider granulometric distribution can amplify the likelihood for crack deflection and increased flexural strength^16,24^.

## CONCLUSION

The strength characteristics of CAD/CAM composites vary according to their composition and microstructure. VE presented the lowest strength and highest Weibull modulus among the materials. LU and CM presented similar strength parameters compared with the direct restorative material tested.
